# Sialylation transmogrifies human breast and pancreatic cancer cells into 3D multicellular tumor spheroids using cyclic RGD-peptide induced self-assembly

**DOI:** 10.18632/oncotarget.11868

**Published:** 2016-09-06

**Authors:** Roman Akasov, Sabah Haq, Fiona Haxho, Vanessa Samuel, Sergey V. Burov, Elena Markvicheva, Ronald J. Neufeld, Myron R. Szewczuk

**Affiliations:** ^1^ Shemyakin-Ovchinnikov Institute of Bioorganic Chemistry, Russian Academy of Sciences, 117997 Moscow, Russia; ^2^ Department of Biomedical and Molecular Sciences, Queen's University, Kingston, Ontario, K7L 3N6 Canada; ^3^ Institute of Macromolecular Compounds, Russian Academy of Sciences, Petersburg, 119004 Russia; ^4^ Department of Chemical Engineering, Queen's University, Kingston, Ontario, K7L 3N6 Canada

**Keywords:** sialylation, spheroids, oseltamivir phosphate, pancreatic cancer, breast cancer

## Abstract

Multicellular tumor spheroids (MTS) have been at the forefront of cancer research, designed to mimic tumor-like developmental patterns *in vitro*. Tumor growth *in vivo* is highly influenced by aberrant cell surface-specific sialoglycan structures on glycoproteins. Aberrant sialoglycan patterns that facilitate MTS formation are not well defined. Matrix-free spheroids from breast MCF-7 and pancreatic PANC1 cancer cell lines and their respective tamoxifen (TMX) and gemcitabine (Gem) resistant variants were generated using the RGD platform of cyclic Arg-Gly-Asp-D-Phe-Lys peptide modified with 4-carboxybutyl-triphenylphosphonium bromide (cyclo-RGDfK (TPP)). MCF-7 and MCF-7 TMX cells formed tight spheroids both in the classical agarose-and RGD-based platforms while all PANC1 cells formed loose aggregates. Using lectin histochemistry staining, sialidase assay, neuraminidase (*Vibrio cholerae*) and oseltamivir phosphate (OP) neuraminidase inhibitor treatments, MCF-7 and PANC1 cells and their drug-resistant variants expressed different sialic acid (SA) content on their cell surfaces. α-2,3- and α-2,6-sialic acid surface residues facilitated spheroid formation under cyclo-RGDfK(TPP)-induced self-assembly. Pretreatment with α-2,3- SA specific *Maackia amurensis* (MAL-II) lectin, α-2,6-SA specific *Sambucus nigra* (SNA) lectin, and exogenous α-2,6-SA specific neuraminidase (*Vibrio cholerae*) dose-dependently reduced spheroid volume. OP enhanced cell aggregation and compaction forming spheroids. PANC1 and MDA-MB231 xenograft tumors from untreated and OP-treated RAGxCγ double mutant mice expressed significantly higher levels of α-2,3- SA over α-2,6-SA. MCF-7 spheroids also expressed a high α-2,3-SA to α-2,6-SA ratio. These results suggest that the relative levels of specific sialoglycan structures on the cell surface correlate with the ability of cancer cells to form avascular multicellular tumor spheroids and *in vivo* xenograft tumors.

## INTRODUCTION

The multicellular tumor spheroid (MTS) is a promising 3D model platform that enables the study of tumor cell development, morphology, cellular motility and drug resistance *in vitro* [1–4]. The MTS mimics the *in vivo* microenvironment which plays a dominant role in multidrug resistance and various cell processes, including epithelial-mesenchymal transition (EMT) and metastasis [5, 6]. MTSs are generally used for novel anticancer drug screening [7, 8]. Since spheroids resemble the 3D architecture of avascular tumors, including multicellular arrangement and extracellular matrix deposition typically found *in vivo*, spheroid cells also demonstrate enhanced resistance to chemotherapy [9]. Tumor spheroids in matrigel or in ECM-based matrixes are good study models to investigate cell motility and anti-metastatic compounds *in vitro* [6, 10]. However, novel MTS formations, particularly under matrix-free conditions, are being developed to study the 3D architecture of avascular tumor models [1, 9, 11–13], especially in relation to metastasis, invasion and therapeutic drug screening [13, 14]. Presently, the molecular development of MTS formation by cancer cells may involve (a) cell surface proteins binding fibronectin which induces 3D cohesion [15], (b) under conditions of random positioning machine (RPM) simulating microgravity, the expression of 28 genes aside from β -tubulin is mutually controlled by a key cytokine interleukin-8 (IL-8 or CXCL8) gene within the framework of 6 extracellular, 6 membrane, 15 cytoplasmic and 2 nuclear proteins [16], and/or (c) the integrins' interactions with the extracellular matrices (ECM) and intracellular components within the cellular cytoskeleton in particular response to mechanical stimulation [16, 17].

It has been reported that MTS formation involves a number of highly glycosylated integrins such as αvβ3 and α5β1 on the cell surface [18, 19]. It is well known that integrin expression correlates with metastases in a large variety of cancers [20]. Since integrins are highly glycosylated receptors, recent reports have reviewed altered expression of sialylated glycoproteins with elevated levels of cell-surface α2,6-sialic acids (SA) that are linked to colorectal cancer metastasis, radio-resistance, and chemoresistance [21, 22]. In addition, the altered mammalian sialidase(s) expression was reported not to result from metastatic potential, but rather from a determining event affecting metastatic ability [23]. It was proposed by the report that SA expression on tumor cell surfaces appears to vary from cell to cell. Other reports have shown that altered sialylation of glycoproteins is closely associated with metastatic potential and cell invasiveness [24–29]. With regard to integrins, Pocheć et al. [30] proposed that the β1-6-branched sialic acid of αvβ3 integrins promotes the metastatic characteristics and migration of melanoma cells.

Recently, we have shown that a synthetic cyclic RGD-peptide induces formation of 3D MTS in a simple, single-step, reproducible procedure. The resulting MTS can be developed and employed as 3D models for assessing antitumor drug efficacy [31] and was studied in twelve cancer cell lines. The report describes the self-assembly of cancer cells from monolayer cultures into MTS, a process that was directly induced by the RGD-peptide. The self-assembly formation of monolayer cultures into MTS was induced by the cyclic Arg-Gly-Asp-D-Phe-Lys (cyclo-RGDfK) peptide, modified with 4-carboxybutyl-triphenylphosphonium bromide cation (TPP). The resulting modified peptide, cyclo-RGDfK(TPP) was used in the concentration range of 10-100 uM. The 3D characterization of the spheroids showed unimodal structures, ranging from 60-120 μm in diameter, and varying between cell lines and medium serum concentration [31]. The report also proposes that these cyclo-RGDfK(TPP) peptides mimick the natural ECM protein's ability to induce cell aggregation via α5β1 integrin.

To evaluate the role of sialylation of cancer cell surfaces in spheroid formation, we used the cyclo-RGDfK(TPP) approach to biochemically induce cell aggregation and compaction, transmogrifying monolayer cancer cells into tumor spheroids.

## RESULTS

### Spheroid formation

**Figure 1:**
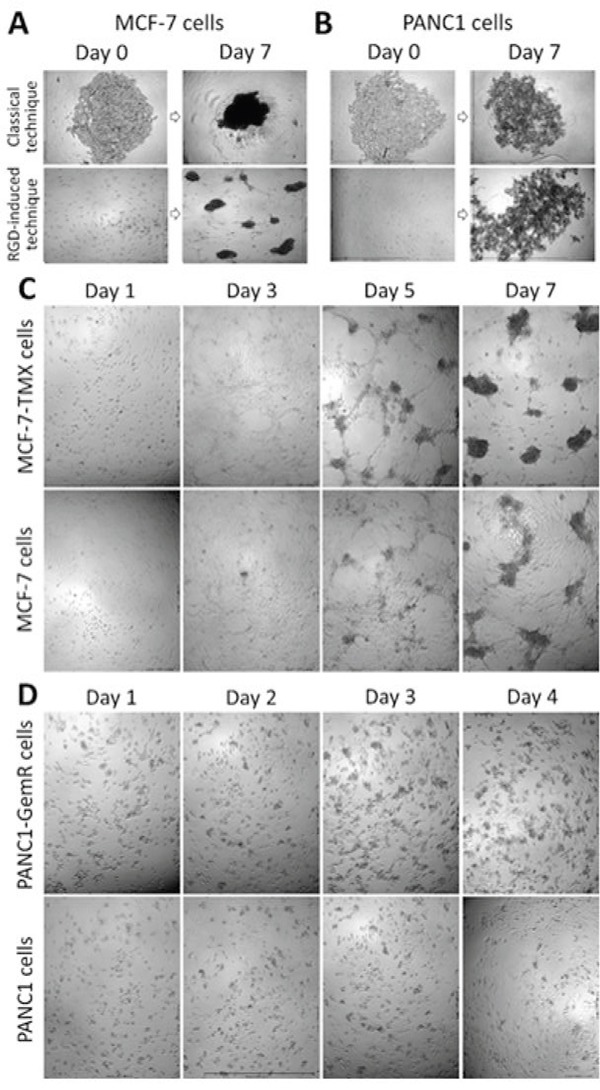
Phase-contrast images of time-dependent spheroid-forming cells derived from MCF-7 A, C. and PANC1 B, D.; 4x objective (A) and (B) spheroid forming cell aggregation on agarose-coated surfaces vs RGD-induced platform using 10,000 cells per well of 96-well plate for 7 days of incubation. The cyclo-RGDfK(TPP) concentration was 25 μM for PANC1 cells and 50 μM for MCF-7 cells. (C) MCF-7 vs MCF-7 TMX and (D) PANC1 vs PANC1-GemR spheroid-forming cell aggregation in the presence of 25 μM (C) and 12.5 μM (D) cyclo-RGDfK(TPP) with 10,000 cells per well, 1-7 days of incubation.

The ability of cancer cells and their chemoresistant variants to form spheroids was studied using the RGD-peptide-based platform which causes specific biochemical alterations of cell surface receptors. These alterations induced self-assembly in monolayer cell cultures into 3D MTS by facilitating cell-cell recognitions, interactions and adhesion [31]. The hypothesis is that the RGD-peptide platform potentiates a higher tendency for cell clustering and compaction. To test this hypothesis, we asked whether the RGD-peptide approach is a universal platform to form tumor spheroids. Here, human breast adenocarcinoma MCF-7 cells formed tight compact spheroids using both the classical and RGD-based platforms (Figures 1A and 1C), while pancreatic carcinoma PANC1 cells formed only loose aggregates even after 7 days of incubation (Figure 1B and 1D). PANC1 cells forming aggregate-like spheroids are consistent with another report using PANC1 cells on tissue culture dishes containing conditioned serum-free medium [32]. We have reported similar aggregate-like irregular spheroids using cyclo-RGDfK(TPP) for malignant melanoma A-375 cells [31].

The ability of MCF-7 and PANC1 cells to form spheroids is proposed here to be dependent on the property of the cell lines, and not on the platform used. Using the cyclo-RGDfK(TPP) peptide approach to form spheroids, tamoxifen-resistant MCF-7 TMX (Figure 1B) and gemcitabine-resistant PANC1-GemR (Figure 1D) cells readily formed spheroids compared with their parental cell lines. The MCF-7 TMX cells formed complete spheroids after 7 days of incubation with 25 μM cyclo-RGDfK(TPP), while parental MCF-7 cells did not form clear-cut spheroids under these conditions (Figure 1C). To generate spheroids from MCF-7 cells, a minimum of 50 μM cyclo-RGDfK(TPP) was needed. For PANC1-GemR cells, the formation of irregular spheroid-like aggregates was observed after 3-4 days of incubation with 12.5 μM of cyclo-RGDfK(TPP) (Figure 1D), while 25 μM peptide was required for parental PANC1. In contrast, cells on agarose-coated plates did not show any differences in spheroid cell formation between MCF-7 and MCF-7 TMX cells (Supplemental Figure 1). Collectively, the results suggest that the chemoresistant cancer cells may have characteristic properties that make them more liable to cell aggregation and compaction using the cyclo-RGDfK(TPP) platform compared with the parental cell lines.

**Figure 2:**
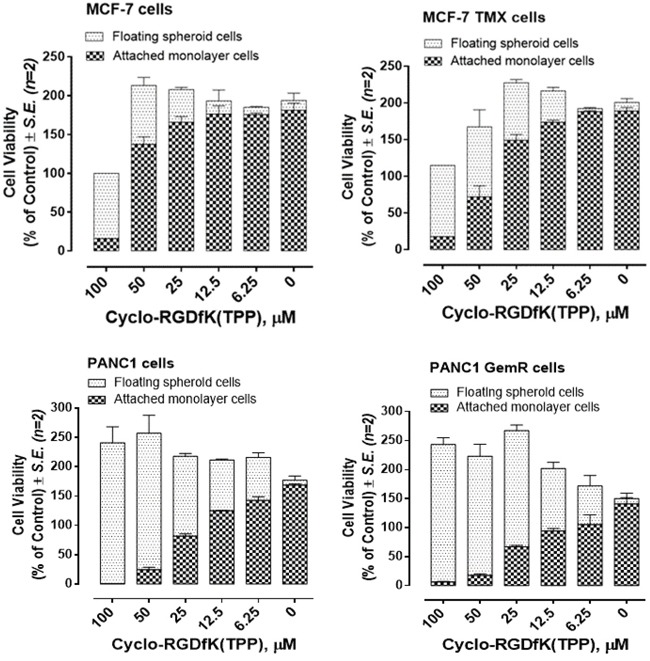
Cell viability of monolayer attached and spheroid-floating MCF-7, MCF-7 TMX, PANC1 and PANC1 GemR cells treated with cyclo-RGDfK(TPP) at indicated doses using modified WST-1 assay Cells were incubated in 96-well plates (5,000 cells/well) and allowed to adhere for 24 hours in 1× DMEM containing 10% FCS. The media were replaced with fresh DMEM containing 5% FCS with or without various concentrations of cyclo-RGDfK(TPP) for 4 days of incubation. Cell viability is expressed as cell viability (% of control) ± *S.E.* of two independent experiments.

Using the cyclo-RGDfK(TPP) peptide platform, we determined the optimal ability of cell aggregation and compaction, leading to the formation of spheroids. Here, the growth of MCF-7, MCF-7-TMX, PANC1 and PANC1-GemR spheroid cells was facilitated by cyclo-RGDfK(TPP) peptide at different concentrations, and performed the cell proliferation assay using the WST-1 reagent [33]. The reagent was added simultaneously to the removed supernatant with floating spheroids and to the residual monolayer cells adherent to the well plate after 4 days of cell growth. Live cell numbers are proportional to absorbance at 420 nm. For MCF-7 TMX cells, more floating spheroid cells and less adherent cells were found compared to MCF-7 cells in the range of 12.5-50 μM cyclo-RGDfK(TPP) (Figure 2). There were no differences found up to 6.25 μM of cyclo-RGDfK(TPP) peptide (no spheroid formation) and for 100 μM of peptide (spheroid cells). Collectively, cyclo-RGDfK(TPP) peptide clearly promoted spheroid formation dose-dependently for both parental and chemoresistant cells. The effects of the peptide in promoting spheroid formation are more robust for the MCF-7 TMX cells than the MCF-7 cells over most of the range of peptide concentrations. The effect of cyclo-RGDfK(TPP) peptide on PANC1-GemR spheroid formation is better in the mid-range of 25 μM of cyclo-RGDfK(TPP) peptide concentration, but the differences are not significant at low and high concentrations of peptide.

### Sialylation of cell surface glycoproteins in multicellular tumor spheroid formation

**Figure 3:**
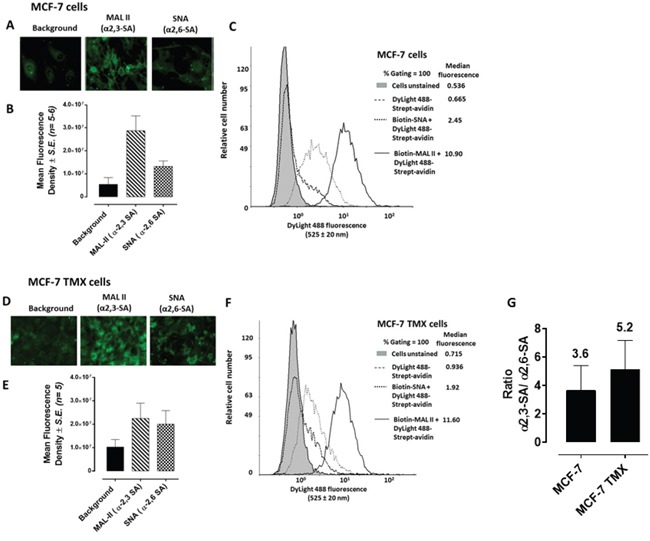
Fluorescent microscopy images of MCF-7 A. and MCF-7 TMX D. cells stained with biotinylated MAL-II and SNA on ice followed with avidin-fluorescein and fixed Background has only the avidin-fluorescein. Stained cells were visualized by epifluorescence microscopy using a x20 objective. **B.** and **E.** Quantitative analysis was done by assessing the density of cell staining corrected for background for 5-6 separate image panels using Corel Photo Paint 8.0 software. Each bar in the graphs represents the mean fluorescence corrected density of staining ± *S.E.* (error bars) for all cells within the respective images. **C.** and **F.** Flow cytometry analysis of biotinylated MAL II or biotinylated SNA staining of cell surface of live indicated cells. Histograms show staining with biotinylated lectins after incubation on ice for 1 hr and followed with DyLight 488 conjugated strept-avidin for additional 30 min on ice. Control cells were stained with DyLight488 conjugated strept-avidin for 30 min on ice. Cells were analyzed by Beckman Coulter Cytomics FC500 flow cytometry and CxP software (Beckman Coulter). Overlay histograms are displayed. Control unstained cells are depicted as gray-filled histogram; DyLight 488 conjugated strept-avidin treated cells, black-dashed unfilled histogram; biotinylated SNA stained cells plus DyLight 488 conjugated strept-avidin, dotted black line unfilled histogram; biotinylated MAL II stained cells plus DyLight 488 conjugated strept-avidin, black line unfilled histogram. The median fluorescence for each histogram is for 500,000 acquired cells (100% gated). **G.** Graph of normalized ratios of α2,3-SA/α2,6-SA to the unstained cells from flow cytometry analyses is displayed. The data are the mean ± *S.E.M.* of 2-3 independent experiments.

**Figure 4:**
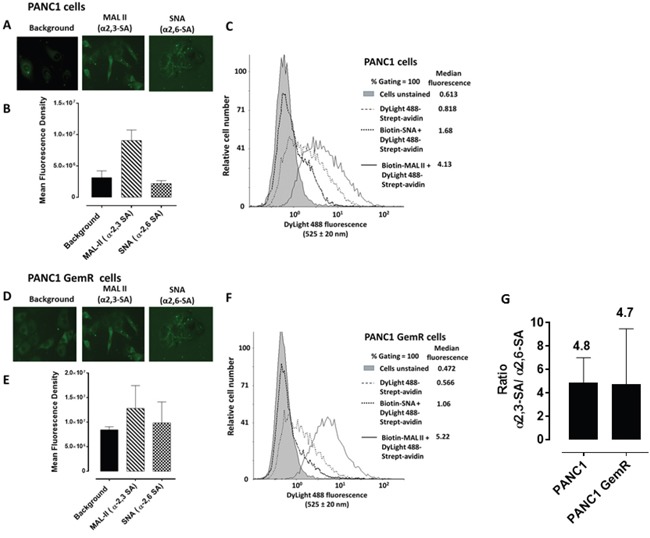
Fluorescent microscopy images of PANC1 A. and PANC1 GemR D. cells stained with biotinylated MAL-II and SNA on ice followed with avidin-fluorescein and fixed Background control has only the avidin-fluorescein. Stained cells were visualized by epifluorescence microscopy using a x20 objective. **B, C, E, F, G.** Quantitative analysis and flow cytometry analyses are similarly described in Figure 3 except for the flow data, where the median fluorescence for each histogram is indicated for 200000 acquired cells (100% gated).

Since MTS and CD24- and CD44-overexpressing cancer stem-like PANC1 cells show high expression of fucosylated glycans using specific lectins binding to the spheroid cells [32], we examined prior to spheroid formation the cell surface sialylation of monolayer MCF-7, MCF-7 TMX cells (Figure 3), and PANC1 and PANC1-GemR cells (Figure 4). This analysis was conducted using lectin histochemistry staining and flow cytometry with α-2,3-sialic acid (SA) specific *Maackia amurensis* (MAL-II) and α-2,6-SA specific *Sambucus nigra* (SNA). The data indicate that the chemoresistant MCF-7 TMX cells (Figure 3G) have more α-2,3-SA cell surface expression than α-2,6-SA compared to the parental cell lines as determined by flow cytometry analyses. The significance of these findings suggest that chemoresistant MCF-7 TMX and PANC1-GemR may have particular sialylated N-linked glycans that contribute to the metastatic phenotype and potential of tumor cells, as proposed by Dennis and Laferte [25], Park and Lee [21] and Haxho et al. [22]. In support of our data, Cui et al. [34] examined the α2,3-SA expression levels in primary and pair-matched lymph node metastatic human tumors as well in triple negative MDA-MB-231, invasive ductal breast carcinoma T-47D and MCF-7 cell lines. Their data showed that the primary lymph node metastatic tumors had significantly elevated levels of α2,3-SA compared to that of primary ones. In addition, the highly metastatic MBA-MB-231 breast cancer cell line also expressed the highest α2,3-SA content compared to other cell lines. These findings were shown to be linked to the α2,3 sialyltransferase mRNA levels.

**Figure 5:**
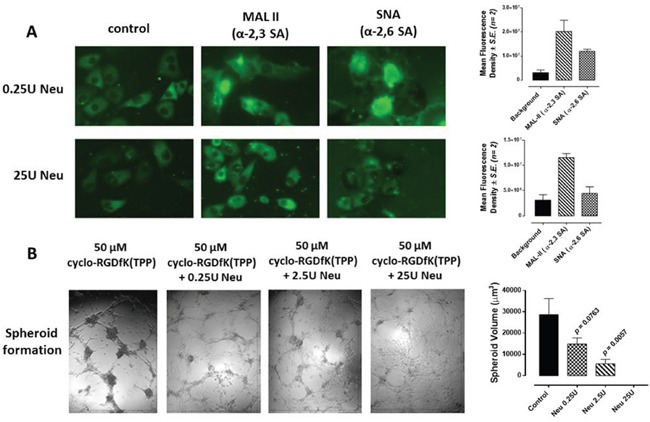
A. Fluorescent microscopy images of MCF-7 stained with biotinylated MAL-II and SNA followed with avidin-fluorescein after 24 h of incubation with 25U or 0.25U of neuraminidase (Neu) (*Vibrio cholerae*, cleavage of α2,6-SA > α2,3-SA > α2,8-SA) Background control has only the avidin-fluorescein. Stained cells were visualized by epifluorescence microscopy using a x20 objective. Quantitative analysis was done by assessing the density of cell staining corrected for background in each panel using Corel Photo Paint 8.0 software. Each bar in the graphs represents the mean corrected density of staining ± *S.E.* (error bars) for all cells within the respective images. **B.** MCF-7 cells were treated with neuraminidase (Neu, *Vibrio cholerae*) at the indicated dosages together with 50 μM cyclo-RGDfK(TPP) with 10,000 cells per well in 96-well plate for 4 days of incubation. Spheroid volume was measured using V = (4/3) π r^3^ where r= average radius (microns) with inverted microscope and 4x objective. Spheroid is defined as a compact rounded spheroid with a distinct border of diameter ≥60 μm containing cells indistinguishable from one another. Each bar in the graph represents mean spheroid volume ± *S.E.* (error bars) for all spheroids within the representative images. Results were compared by a one-way ANOVA at 95% confidence using Fisher's LSD test. Data are a representation of one out of three independent experiments showing similar results.

Other reports have shown that the catalytic cleavage of SA of E-cadherin on the cell surface of MCF-7 cells by neuraminidase (from *Vibrio cholerae*, cleavage rate of α2,6-SA > α2,3-SA > α2,8-SA) prevented cell adhesion or aggregation [35]. We hypothesized that the cell surface expression of α2,6-SA facilitates spheroid formation. Here, pretreatment of MCF-7 cells with a neuraminidase (from *Vibrio cholerae*) was performed followed with MAL-II and SNA lectin histochemistry staining. Treatment of MCF-7 cells with 25U of neuraminidase for 24 hrs showed a pronounced removal of α2,6-SA compared with no effects on α2,3-SA (Figure 5A). The data in Figure 5B clearly support the hypothesis. MCF-7 cells treated with 0.25U or 25U neuraminidase in the presence of 50 μM cyclo-RGDfK(TPP) significantly reduced spheroid volume compared with the untreated control. To measure spheroid volume, we defined a spheroid as a compact rounded aggregation of cells with a distinct border of diameter ≥60 μm containing cells indistinguishable from one another, according to Akasov et al. [31].

**Figure 6:**
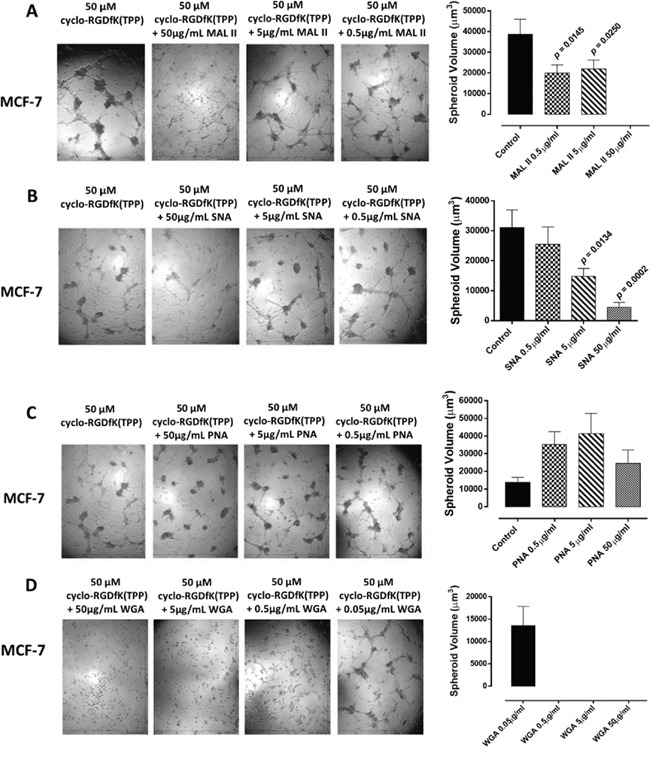
MCF-7 cells were treated with A. *M. amurensis* lectin MAL II (α2,3-SA), B. *Sambucus nigra* lectin SNA (α2,6-SA), C. peanut agglutinin PNA (specific for galactosyl (β-1,3) N-acetylgalactosamine) or D. wheat germ agglutinin WGA (N-acetylglucosamine residues) at the indicated dosages together with 50 μM cyclo-RGDfK(TPP) and 10,000 cells per well in 96-well plate for 4 days of incubation Spheroid volume was measured using V = (4/3) π r^3^ where r = average radius (microns) with inverted microscope and 4x objective. Each bar in the graph represents mean spheroid volume ± *S.E.* (error bars) for all spheroids within the representative images. Results were compared by a one-way ANOVA at 95% confidence using Fisher's LSD test. Data are a representation of one out of three independent experiments showing similar results.

To test the role of SA on spheroid formation, we performed a lectin inhibition assay where monolayers of MCF-7 cells were treated with *M. amurensis* lectin MAL II (α2,3-SA), *Sambucus nigra* lectin SNA (α2,6-SA), peanut agglutinin (PNA, galactosyl (β-1,3) N-acetylgalactosamine), or wheat germ agglutinin (WGA, N-acetylglucosamine residues) in a dose-dependent manner together each with 50 μM cyclo-RGDfK(TPP) for 4 days of incubation. Pretreatment of MCF-7 cells with MAL II and SNA lectins dose-dependently and significantly reduced spheroid formation in the presence of 50 μM cyclo-RGDfK(TPP) (Figure 6A and 6B). In contrast, PNA and WGA had no inhibitory effects on cyclo-RGDfK(TPP)-induced MCF-7 spheroid formation (Figure 6C and 6D). It is noteworthy that WGA was shown to have an IC_50_ of 5.4 μg/mL for monolayer MCF-7 cells based on the cell viability assay (data not shown), which is consistent with another report [36].

We have recently shown activation of epidermal growth factor receptors (EGFR) is regulated by a cell-surface signaling complex involving neuraminidase-1 (Neu1), matrix metaloproteinase-9 (MMP9), and the neuromedin B G protein-coupled receptor (GPCR) [37]. The report also provided evidence to indicate that Neu1 specifically cleaves α-2,3-SA on EGF receptors to remove steric hindrance of the receptor, with subsequent receptor association, activation and downstream signaling. Neu1 complexed with MMP-9 and GPCR tethered to receptor tyrosine kinases (RTKs) and TOLL-like receptors (TLRs) has been reported by us as a major target in multistage tumorigenesis [22]. Furthermore, Neu4 sialidase has been reported to be a regulator of neurospheres derived from patient samples and glioblastoma multiforme (GBM) cell line [38]. The report provided strong evidence to demonstrate that Neu4 overexpression is associated with an upregulation of stem cell-like properties in neuroblastoma cells. Remarkably, Neu4 expression levels were significantly decreased in differentiated and non-neurosphere GBM cells.

**Figure 7:**
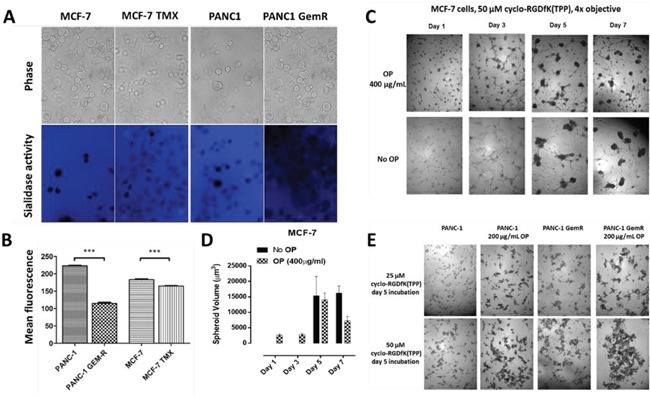
**A.** Sialidase activity in indicated live cells. Cells were allowed to adhere on 12 mm circular glass slides in media containing 10% fetal calf sera for 24 h After removing medium, 0.318 mM 4-MUNANA (4-MU) substrate (2′-(4-methlyumbelliferyl)-α-N-acetylneuraminic acid) in Tris-buffered saline (pH 7.4) was added to live cells alone. The substrate was hydrolyzed by sialidase enzymes expressed on cell surface to give free 4-methylumbelliferone, which has a fluorescence emission at 450 nm (blue color) following excitation at 365 nm. Fluorescent images were taken at 1-2 min after adding substrate using epi-fluorescent microscopy (10× objective). **B.** The mean fluorescence of 50 multi-point replicates was quantified using the Image J software. Results were compared by a one-way ANOVA at 95% confidence using t-test. Data are a representation of one out of three independent experiments showing similar results. **C.** MCF-7 spheroid formation in the presence of 50 μM of cyclo-RGDfK(TPP) with 400 μg/mL OP or without (no inhibitor) with 10,000 cells per well in 96-well plate for 1-7 days of incubation. **D.** Spheroid volume was measured using V = (4/3) π r^3^ where r= average radius (microns) with inverted microscope and 4x objective. Each bar in the graph represents mean spheroid volume ± *S.E.* (error bars) for all spheroids within the representative images. **E.** PANC1and PANC1-GemR cell aggregation and compaction in the presence of 25 μM or 50 μM of cyclo-RGDfK(TPP) with or without 200 μg/mL OP, 5 days of incubation.

If activated Neu1 and Neu4 in complex with glycosylated receptors act as promoters of spheroid formation, it is important to assess the sialidase activity of live cells in both monolayer cultures, as well as MTS. In this report, we conducted a live cell sialidase assay [39–41] to determine whether the selected cancer cell lines express sialidase activity on their surface. Naïve live MCF-7 and PANC1 cells exhibited significantly more sialidase activity than the chemoresistant variants, MCF-7 TMX and PANC1-GemR (Figure 7A). Mean fluorescence surrounding viable cells is depicted in the bar graph (Figure 7B). Collectively, sialidase activity reduction on the surface of chemoresistant MCF-7 TMX and PANC1-GemR cells identifies them as non-sphere-differentiated chemoresistant cells, similar to previous reports for non-neurosphere-differentiated GBM cells [38].

We have also reported that oseltamivir phosphate (OP) inhibits Neu1 [41] and Neu4 [42] sialidase activity with IC_50_ values of 1.175μM for LPS-stimulated BMC2 macrophage cells and 0.019μM for thymoquinone (TQ)-stimulated BMC2 cells, respectively. TQ specifically activates Neu4 sialidase [38, 42, 43]. Here, we used MCF-7, PANC1 and PANC1-GemR cells to determine if OP-inhibition of sialidase activity is involved in cyclo-RGDfK(TPP)-facilitated spheroid formation. MCF-7 cells treated with OP (400 μg/mL) and cyclo-RGDfK(TPP) (50μM) produced early visible spheroids after 1-3 days and an increase in spheroid volume by day 5 (Figure 7B and 7C). There was a decrease in spheroid volume by day 7 in the presence of OP. For OP ≥ 400 μg/mL, the cell viability of monolayer PANC1 cells [44], MCF-7 and tamoxifen-resistant MCF-7 TMX cells [45] was significantly reduced after 3 days. For PANC1 and PANC1-GemR cells, 200 μg/mL OP and 50μM cyclo-RGDfK(TPP) for 5 days produced cell aggregates and compaction that were darker than those without OP, indicating improved cell compaction and tighter cell-cell contacts (Figure 6B). The effect of OP on spheroid formation might be explained by (a) OP increases the expression of E-cadherin [44], an important role in spheroid compaction of breast carcinoma [46], hepatoma [47] and hepatocytes [48, 49] and/or (b) inhibition of Neu4 sialidase [38].

### Sialylation of cell surface glycoproteins in xenograft tumors of PANC 1 and MDA-MB231 cancer cells in mouse models of human disease

**Figure 8:**
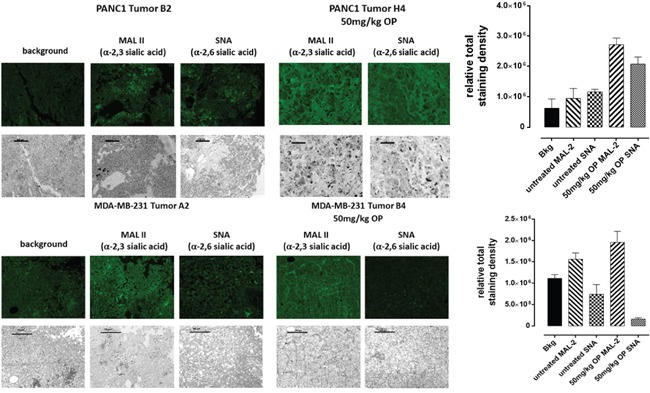
Fluorescence histochemical detection of α-2,3 SA and α-2,6 SA expressions in paraffin-embedded tumor tissues archived from xenograft tumors of PANC 1 and MDA-MB231 cells growing in RAG xCγ double mutant mice Mice were implanted with 1 × 10^6^ PANC1 or MDA-MB231 cells cutaneously on the rear flank and OP treatment at indicated dosages began at 22–23 days post implantation when tumors reached 100–200 mm3. Paraffin-embedded tumor sections (5 μm) on glass slides were processed for lectin histochemistry using biotinylated MAL II and SNA followed with avidin fluorescein and fluorescence mounting media. Background control sections (Bkg) were prepared without the biotinylated lectins. Tissue sections were visualized and photographed using a Zeiss Imager M2 fluorescence microscope at 200× magnification. Images are representative of at least five fields of view from two tumor sections.

**Figure 9:**
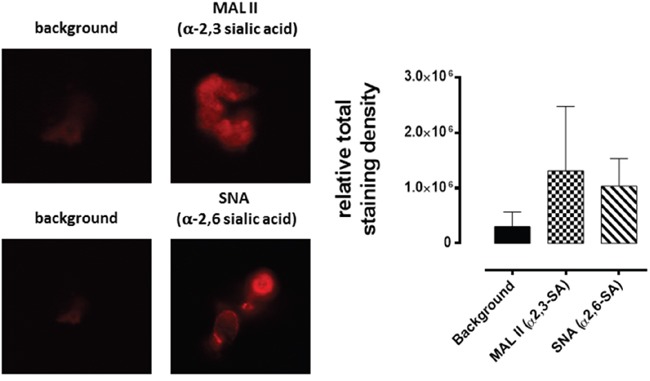
Fluorescence cytochemical detection of α-2,3 SA and α-2,6 SA expressions in MCF-7 spheroids Spheroids on glass slides were processed for lectin histochemistry using biotinylated MAL II and SNA followed with streptavidin DyLight 594 and fluorescence mounting media. Background control sections (Bkg) were prepared without the biotinylated lectins. Spheroids were visualized and photographed using a Zeiss Imager M2 fluorescence microscope at 200× magnification. Images are representative of at least five fields of view.

Levels of α2,6-SA and α2,3-SA expression were analyzed in tumor-derived sections of PANC1 and MDA-MB231 xenografts growing in RAGxCγ double mutant mice. Cohorts were dosed with 50 mg/kg oseltamivir phosphate (OP) for PANC1 tumors or 30 mg/kg OP for MDA-MB231 tumors, and were analyzed in comparison to their respective, untreated cohorts. Since OP affects α2,3-SA expression in PANC1 and MCF-7 cells, paraffin-embedded necropsy tumor sections were investigated for differences in α2,6-SA and α2,3-SA expression levels. Using fluorescence histochemical staining, tumors from OP-treated cohort showed strong staining for α2,3-SA compared with the untreated cohort (Figure 8). Sections with no primary antibody added (background fluorescence control) demonstrated minimal non-specific staining. Our findings also show that avascular MCF-7 tumor spheroids express higher α2,3-SA over α2,6-SA (Figure 9). Collectively, these data implicate a novel role for cell-surface sialylation, in relation to both sialic acid content and linkage specificity, in the formation of multicellular tumor spheroids.

## DISCUSSION

MCF-7 and PANC-1 cells, and their drug-resistant cancer cell lines (MCF-7 TMX, PANC1-GemR) express different SA content, which influenced their ability to form spheroids under cyclo-RGDfK(TPP)-induced self-assembly. Cancer cell aggregation and compaction correlates with the presence of α-2.3- and α-2,6-sialic acid cell surface residues to form spheroids under cyclo-RGDfK(TPP)-induced self-assembly. Removal or blockage of SA inhibited cell aggregation for MCF-7 and MCF-7 TMX cells under cyclo-RGDfK(TPP) peptide. Neuraminidase inhibitor oseltamivir phosphate enhanced cell aggregation and promoted compaction of cell aggregates.

Using the classical agarose-coated plates to form spheroids in a time-dependent manner, spheroid-forming MCF-7 vs MCF-7 TMX cells and PANC1 vs PANC-GemR cells did not show any differences in spheroid cell formation (Supplemental Figure 1). In the classical platform of spheroid formation, including agarose-coated wells, hanging drop, and rotary culture, the resulting cell aggregation and compaction occur due to acting mechanical forces and/or surface properties which may prevent cell attachment to the cellular matrix in the culture well plate. To form spheroids, all of the cells are required to be in a 3D configuration. Raghavan et al. [1] have recently compared different methodologies to generate tumor spheroids in terms of morphology, cellular arrangement, and chemosensitivity. These methods include hanging drop array plates, liquid overlay on ultra-low attachment plates, and liquid overlay on ultra-low attachment plates with rotating mixing (nutator plates). They found that the different methods of tumor spheroid generation resulted in varied cellular arrangement and chemosensitivity. For most currently available 3D classical methods for spheroid formation, these techniques are time consuming, lack reproducibility and mainly rely on microscopic observations, thus there is a need to standardize and develop rapid protocols for tumor spheroid formations [50].

Terao et al. [32] demonstrated that the gemcitabine-resistant pancreatic cancer cells (PANC1-RG) contained a greater population of cells with stem cell-like properties, when compared to the parental PANC1 cells. Specifically, the stem cell-like phenotype was characterized by higher levels of both CD24 and CD44 in the PANC1-RG cells. The same group also found that PANC1-RG cells could form spheroids more effectively, and larger in size, than their PANC1 counterparts. It is noteworthy that a few studies using spheroid formation on chemoresistant cancer cells have been performed [14, 50]. It is interesting to note from the report by Terao et al. [32] that sphere-forming cells and PANC1 stem-like cells expressed high levels of fucosylated glycans. Using AAL and AOL lectins in western blot analysis, it was found that spheroid-forming cells demonstrated increased binding to both lectins, when compared to the monolayer cells [32]. These findings may be partially explained by the branched N-glycans, including fucosylation sites, covalently conjugated to cell adhesion molecules. These glycans, linked to both cadherins and integrins, are known to play key structural and functional roles in cell-cell recognition and interaction [51].

Consistent with this premise, several other reports have shown that breast carcinoma cell lines [46], hepatoma [47] and hepatocytes [48, 49] are able to form spheroids dependent on E-cadherin. Iglesias et al. [46] analyzed the capability of 11 breast cancer cell lines to form mammospheres, and thus able to continually proliferate in a non-adherent state. Only MCF7, T47D, BT474, MDA-MB-436 and JIMT1 cells could be passaged and developed into mammospheres, while other cancer cells including SKBR3, MDA-MB-231, MDA-MB-468 and MDA-MB-435 could only form temporary aggregates, with dramatic reduction in cell viability upon a second passage.

The previously described report by Silvestri et. al [38] demonstrated that glioblastoma stem cells (GSC), isolated from patients and multiforme cell lines, are associated with an upregulation of Neu4 activity. The catalytic activity of Neu4 has been shown to trigger key events in glioblastoma stem cells, including (a) glycogen synthase kinase 3B (GSK3b) activation, (b) subsequent suppression of Sonic Hedgehog and Wnt/B-catenin signaling, (c) downregulation of stem cell-like gene expression and biomarkers (characterized by a significant decrease in NANOG, OCT-4, SOX-2, CD133, ganglioside GD3, and aberrant protein glycosylation patterns), and (d) a marked reduction in GSC viability. TQ-induced Neu4 activity can target plasma membrane-bound sialylated receptors such as TOLL like receptor 4 (TLR4) [42]. The proposed mechanism occurs through a cell-surface signaling axis consisting of MMP-9 and neuromedin B GPCR, which form complexes with TLR4 to generate a functional receptor. Collectively, the inhibition of Neu4 and/or Neu1 with OP is proposed to affect the sialylation of glycoproteins and glycolipids on the cell surface. This results in a glycome phenotypically similar to that of differentiated cells, and thus reducing the stem cell-like glycosylation phenotype.

The potential clinical implications of these studies suggest that selectively targeting Neu4 and Neu1 could modify the glycosylation state of cell surface proteins, thus reducing tumor growth and preventing metastatic disease. Preclinical data from our studies reveal significant and reproducible *in vitro* and *in vivo* results that OP therapy decreases EGF receptor over-activity and downstream signaling in cancer cells and in tumor lysates obtained from heterotropic xenografts of MiaPaCa-2 pancreatic tumors growing in RAGxCγ double mutant mice [37]. OP therapy also impeded tumor growth, blocks tumor neovascularization and metastases. Collectively, we propose here that Neu1 is a novel alternate candidate target using OP therapy in restraining the growth, metastases, tumor neovascularization as well as macrophage-mediated tumorigenesis of human cancers. Here, Neu1 forms a complex with a broad range of glycosylated growth factor receptors including extracellular and intracellular TOLL-like (TLR) sensing receptors, as reviewed by Haxho et al [22].

In conclusion, tumor spheroids have emerged and show promise as a model for investigating growth and development of tumors, cancer cell motility, and drug efficacy. The present report provides evidence for the important role of specific sialoglycan structures expressed on cancer cells to form avascular multicellular tumor spheroids and *in vivo* xenograft tumors. Future studies should build upon these findings and explore alternate and novel methods to target the cancer cell glycome and the unique sialylation patterns of the adhesion molecules involved in spheroid formation and tumor progression.

## MATERIALS AND METHODS

### Reagents

*Maackia amurensis* lectin II (MAL II) was purchased from Sigma. *Sambucus nigra* lectin (SNA), Peanut Agglutinin (PNA) and Wheat Germ Agglutinin (WGA) was bought from Vector Laboratories. Arg-Gly-Asp-D-Phe-Lys (cyclo-RGDfK) modified with 4-carboxy-butyl-triphenylphosphonium bromide (cyclo-RGDfK(TPP)) peptide was synthesized by Prof. Dr. S. Burov, Saint-Petersburg, Russia and further characterized by us [31].

Neuraminidase from *Vibrio cholerae* was obtained from GIBCO laboratories. Neuraminidase hydrolyzes terminal N- or O-acylneuraminic acids which are α2,6-, α2,3-, or α2,8-linked (rate: α2,6 > α2,3 > α2,8) to oligosaccharides, polysaccharides, mucopolysaccharides, glycoproteins, and glycolipids. One unit is the enzyme activity that hydrolyzes 1 μmol N-acetyl-neuraminosyl-D-lactose within 1 min at 37°C under the following incubation conditions: 10 mM N-acetyl-neuraminosyl-D-lactose, 50 mM sodium acetate, 4 mM calcium chloride, bovine serum albumin, 100 μg/ml, pH 5.5. Specific activity of neuraminidase (*Vibrio cholera*) is 1 μmol N-acetylneuraminic acid per min is split off from human acid α1-glycoprotein (10 mg/ml incubation mixture) by 1 U neuraminidase.

### Cell lines

Human cell lines (breast adenocarcinoma MCF-7 (ATCC ® HTB-22™)) and pancreatic carcinoma PANC1 (ATCC® CRL-1469™) were purchased from ATCC (Manassas, VA 20110 USA) collection. The cells were grown in 1×Dulbecco's Modified Eagle's Medium (DMEM; Gibco, Rockville, MD, USA) conditioned medium, supplemented with 10% fetal calf serum (FCS; HyClone, Logan, UT, USA), and 5 μg/mL plasmocin™ (InvivoGen, San Diego, CA, USA) in a 5% CO_2_ incubator at 37°C. At ~80% confluence, the cells were passaged at least five times before use in the experiments. Tamoxifen-resistant MCF-7 TMX and gemcitabine-resistant PANC1-GemR cells were cultured in media containing 10 μM tamoxifen and 0.01 μM gemcitabine, respectively, for over one year, authenticated in Szewczuk's lab.

### Fluorescent lectin cytochemistry for sialic acid (SA) expression

Cells were seeded and grown overnight on 12 mm circular glass slides in conditioned medium in a sterile 24-well tissue culture plate for 24 hours. Cells were washed with PBS and fixed with 4% paraformaldehyde (PFA) for 20 min or cold methanol (20 min). Following fixation, cells were washed with 1x phosphate-buffered saline pH 7.4 (PBS) and stained with indicated lectins. SNA (biotinylated elderberry bark lectin, B-1305, Vector Laboratories) and MAL II (biotinylated *Maackia amurensis* lectin II, B-1265, Vector Laboratories) at concentrations of 10 μg/mL in PBS were incubated on slides for 1 hr at room temperature. Stained slides were then washed twice with PBS and incubated 1 hr with avidin-fluorescein (10 μg/mL). Cells without biotinylated lectins were used as controls. After three washings with PBS, slides were mounted in cell mounting media and analyzed with Carl Zeiss Imager 2 fluorescence microscope.

### Sialidase assay

Cells were grown overnight on 12 mm circular glass slides in conditioned medium in a sterile 24-well tissue plate. Before the experiment, the cells were incubated in starvation (serum-free) medium for two hours to reach the baseline level of sialidase activity in each cell line. The media was removed and 0.318 mM 4-MUNANA (2′-(4-methylumbelliferyl)-α-D-N-acetylneuraminic acid; Biosynth Intl.) substrate in Tris-buffered saline (TBS, pH 7.4) was added to each well. The substrate was hydrolyzed by sialidase to give free 4-methylumbelliferone, which has a fluorescence emission at 450 nm (blue color) following an excitation at 365 nm. Fluorescent images were taken after 1–2 min using epi-fluorescent microscopy (40× objective). The mean fluorescence surrounding the cells was quantified using the Image J program.

### WST-1 assay

The WST-1 assay, a measure of cell viability based on the reduction of a tetrazolium compound to a soluble derivative [33], was used. The absorbance recorded at 420 nm is directly proportional to the number of living cells in culture. At 80–90% confluence, floating spheroids and attached cells were added to 96-well microwell plates at a density of 5,000 cells/well and incubated overnight. The cells were left untreated as controls for 4 days. Absorbance readings were taken by adding WST-1 (Roche Diagnostics Division de Hoffman La Roche Limitée, Laval-des-Rapides, QC, Canada) as a cell proliferation reagent to each well (10% WST-1 in Dulbecco's Modified Eagle's Medium), followed by incubation at 37°C for 2 hours before reading at the indicated time point. Cell viability was presented as a percentage of control, and illustrated as a bar graph using GraphPad Prism software (GraphPad Software, La Jolla, CA, USA). The following formula was used to determine cell viability as a percent of control for each peptide concentration, where day 0 is when peptide was added:

[(Absorbance of cells in given concentration of peptide)−(Media absorbance)] /

[(Absorbance of cells alone on day 0)−(Media absorbance)] × 100

### Flow cytometry of cell surface SA in live cells

Flow cytometry analysis of biotinylated MAL II or biotinylated SNA was conducted through staining of live cell surfaces. Live cells were stained with biotinylated lectins after incubation on ice for 1 hr and followed with DyLight 488 conjugated strept-avidin for additional 30 min on ice. They were then fixed. Control cells were stained with DyLight488 conjugated strept-avidin for 30 min on ice. Cells were analyzed by Beckman Coulter Cytomics FC500 flow cytometry and CxP software (Beckman Coulter) in the Queen's University Biomedical Imaging Center (QUBIC), Faculty of Health Sciences. Overlay histograms are displayed. Control unstained cells are depicted as gray-filled histogram. DyLight 488 conjugated strept-avidin treated cells are represented by the black-dashed unfilled histogram. Biotinylated SNA stained cells plus DyLight 488 conjugated strept-avidin are depicted by unfilled histogram with the dotted black line. Biotinylated MAL II stained cells plus DyLight 488 conjugated strept-avidin are depicted by unfilled histogram with the black line. The median fluorescence for each histogram is indicated for 500,000 acquired cells (100% gated). The data are a representation of one out of two experiments showing similar results.

### Agarose-based classical technique of spheroids formation

Tumor spheroid formation on agarose-coated plates was performed as previously described [52]. Briefly, 1.5% wt of agarose in 1xDMEM was heated on water bath for 15 min. 50 μL of agarose gel was added to each well of a flat-bottom 96-well plate under sterile conditions. Plates with agarose were cooled down to room temperature for 15 minutes. Cells were seeded on agarose-coated plates (5,000-10,000 cells/well, 100 μL of media in each well) and incubated at 37°C for 1-7 days.

### RGD peptides-based technique of spheroid formation

Spheroid formation was carried out as previously described by us [31]. Briefly, cells were seeded in a flat-bottom 96-well plate (5,000-10,000 cells/well, 100 μL/well) and incubated at 37°C for 2-3 h until the cells attached to the plate bottom. The medium was replaced with 100 μL of 1xDMEM containing a cyclo-RGDfK(TPP) peptide (25-50 μM). The plate was incubated at 37°C for additional 1-7 days.

### Measurement of spheroid volume

Spheroid volume was determined from images obtained with an inverted microscope using 4x or 10x objectives and defined as a compact rounded spheroid with a distinct border of diameter ≥60 μm containing cells indistinguishable from one another. The following formulae were used to determine spheroid volume:

10x objective images: V = (4/3) π r^3^ where r= average radius (microns)

4x objective images: V = (2.5)(4/3) π r^3^ where r= average radius (microns)

For the 4x objective images, the formula includes 2.5 to normalize values to the 10x objective images.

### Lectin histochemistry staining of archived xenograft tumors

Lectin histochemistry staining was used to determine the presence of the characteristic α-2,3-SA and α-2,6-SA expression in human pancreatic PANC1 and triple negative breast MDA-MB231 tumors removed at necropsy from tumor-bearing RAG2xCγ double mutant mice, which had received various treatments (oseltamivir phosphate 50 mg/kg for PANC1 and 30 mg/kg for MDA-MB231 or untreated). Archived processed tumors embedded in paraffin blocks were obtained from previous experiments. An immunodeficient mouse model with a double mutation in RAG2 and Cγ was used as a xenograft mouse model of human pancreatic and breast cancers, as previously reported by our group [45]. The RAG2xCγ double mutant mice were implanted cutaneously with 1×10^6^ cells and OP treatment was started 10 days post-implantation for MDA-MB231 [45] and 16 days for PANC1. Treatment was continued until the mice either died or were euthanized at the end of the experiment. Tumor sections (5 μm) were deparaffinized, heated for 10 minutes in citrate buffer for antigen retrieval, rinsed three times in phosphate-buffered saline, and blocked in 1% bovine serum albumin (Fisher Scientific Company) for 2 hours. Sections were then incubated with biotinylated MAL II or biotinylated SNA overnight, followed by incubation with avidin fluorescein for two hours. Coverslips were added using DAKO fluorescent mounting medium. Background control sections were also prepared in the same way as above, without biotinylated lectins. Tissue sections were visualized and photographed using a Zeiss Image M2 microscope at 200× magnification.

## SUPPLEMENTARY MATERIALS FIGURE


