# Supplementary material for: Sialylation transmogrifies human breast and pancreatic cancer cells into 3D multicellular tumor spheroids using cyclic RGD-peptide induced self-assembly
Source: Oncotarget. 2016 Sep 6;7(40):66119–34. doi: 10.18632/oncotarget.11868 (PMC5323220; doi:10.18632/oncotarget.11868)
Supplement: Supplementary file 1 [file oncotarget-07-66119-s001.pdf]

Sialylation transmogrifies human breast and pancreatic cancer cells into 3D multicellular tumor spheroids using cyclic RGD-peptide induced self-assembly

SUPPLEMENTARY FIGURE

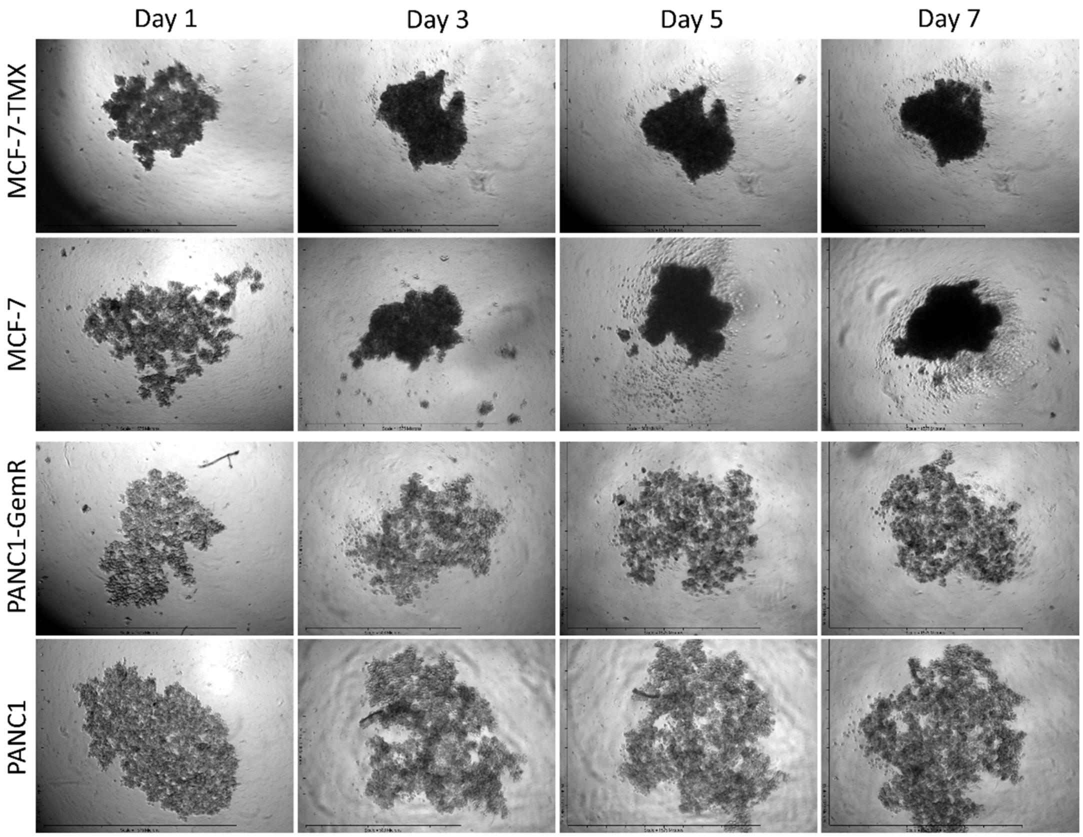

Supplementary Figure S1: Time-dependent spheroid forming MCF-7 vs MCF-7 TMX cells, and PANC1 vs PANC-GemR cells on agarose-coated plates for 1-7 days of incubation, 10,000 cells per well of 96-well plate, 1-7 days of incubation.
